# Patterned Carboxymethyl-Dextran Functionalized Surfaces Using Organic Mixed Monolayers for Biosensing Applications

**DOI:** 10.1021/acsabm.2c00311

**Published:** 2022-06-25

**Authors:** Elena Ambrosetti, Martina Conti, Ana I. Teixeira, Simone Dal Zilio

**Affiliations:** † Department of Medical Biochemistry and Biophysics, Karolinska Institutet, Stockholm 171 77, Sweden; ‡ CNR-IOM, Istituto Officina dei Materiali-Consiglio Nazionale delle Ricerche, Basovizza, 34149 Trieste, Italy

**Keywords:** patterned surfaces, protein anchoring, carboxymethyl-dextran, binding bioassay, surface plasmon resonance

## Abstract

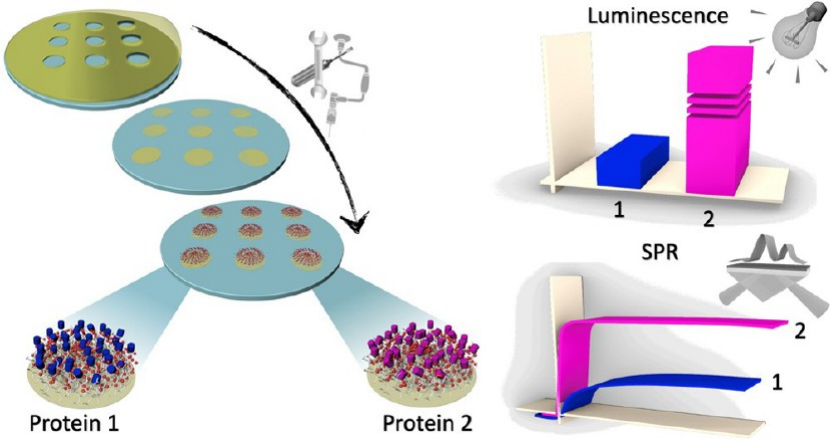

The deposition of biomolecules on biosensing surface platforms plays a key role in achieving the required sensitivity and selectivity for biomolecular interactions analysis. Controlling the interaction between the surface and biomolecules is increasingly becoming a crucial design tool to modulate the surface properties needed to improve the performance of the assay and the detection outcome. Carboxymethyl-dextran (CMD) coating can be exploited to promote chemical grafting of proteins, providing a hydrophilic, bioinert, nonfouling surface and a high surface density of immobilized proteins. In the present work, we developed and optimized a technique to produce a cost-effective CMD-based patterned surface for the immobilization of biomolecules to be used on standard protocols optimization. They consist of silicon or glass substrates with patterned bioactive areas able to efficiently confine the sampling solution by simply exploiting hydrophilic/hydrophobic patterning of the surface. The fabrication process involves the use of low-cost instruments and techniques, compatible with large scale production. The devices were validated through a chemiluminescence assay we recently developed for the analysis of binding of DNA nanoassemblies modified with an affinity binder to target proteins immobilized on the bioactive areas. Through this assay we were able to characterize the chemical reactivity of two target proteins toward a dextran matrix on patterned surfaces and to compare it with model CMD-based surface plasmon resonance (SPR) surfaces. We found a high reproducibility and selectivity in molecular recognition, consistent with results obtained on SPR sensor surfaces. The suggested approach is straightforward, cheap, and provides the means to assess patterned functionalized surfaces for bioanalytical platforms.

## Introduction

Analysis of biomolecular interactions, such as protein–protein, protein–ligand, and protein–nucleic acid interactions, is crucial in many disciplines, such as biochemistry, biotechnology, and medicine. Advances in biomolecular interactions assays have been directed toward improving accuracy, speed, and the ability to screen multiple analytes in parallel.^1^ Surface immobilization of proteins is commonly used in protein biorecognition assays because it allows for easy separation of molecules bound to a protein of interest from unbound molecules, overcoming difficulties in discriminating between them in solution. Another advantage of surface immobilization is the possibility to tune the parameters of the immobilization process, and to control the localization and stability of the molecules. Protein microarrays are miniaturized devices for detection of interactions between proteins and soluble molecules, consisting of a solid surface onto which different proteins are immobilized in discrete spatial locations, forming a protein dot matrix.^2,3^ Enzyme-linked immunosorbent assay (ELISA) is a commonly used solid-phase approach for protein biorecognition that can be implemented as a microarray to yield highly sensitive and specific analyses. However, this assay has several disadvantages: large sample volumes are usually required (low-volume assay formats often require automated and expensive equipment) and cross-reactivity can occur with the secondary antibody, resulting in nonspecific signals. Label-free detection methods have also been investigated for protein microarrays. Mass spectrometry-based methods, for example, have been used for detecting ligands bound to individual proteins printed on protein microarrays. Another powerful surface technology is surface plasmon resonance (SPR) that allows one to analyze label-free biomolecular interactions in real-time.

Irrespective of the analysis approach, the performance of a surface bioassay is strictly dependent on the quality of the biofunctional coating onto the surface. The immobilization of the proteins on the surface can affect protein functionality and the subsequent assay outcome. Therefore, controlling the interaction between the surface and biomolecules is increasingly becoming a crucial design tool to improve assay performance.^4^ Two main strategies are exploited for the attachment of proteins to the solid substrate: physical adsorption and chemical binding. In physical adsorption, proteins spontaneously react with the surface through noncovalent interactions. Some proteins, especially those with intrinsically low internal stability, can undergo conformational changes when physisorbed on surfaces.^5^ In general, predicting the interaction and the subsequent bioactivity of proteins on solid surfaces is challenging, because several molecular phenomena are involved, such as adsorption, desorption, conformational changes, and rearrangements because of the buffer changes required during a typical binding assay.^5^ On the contrary, chemical immobilization entails the introduction of chemical functional groups on a solid surface, through which biomolecules can be covalently immobilized in a site-specific manner, resulting in stronger bonds with the substrate. A powerful strategy to improve assay sensitivity is the use of polymers as an interlayer when immobilizing biomolecules onto a surface, which can facilitate the preservation of protein integrity.^6^ Moreover, polymer spacers can also prevent nonspecific adsorption of proteins onto synthetic surfaces, which lead to a loss in specificity of the assay.^4^ High molecular weight, branched polymers such as hydrogels have more functional groups available for biomolecular interactions than linear polymeric spacers, which contain a few binding sites, and can therefore accommodate a greater number of biomolecules. This, as a result, leads to an improved performance of the assay and a lower limit of detection.^7^ Hydrogels are increasingly becoming a functionalization tool for biosensor applications;^8−11^ their attractiveness is related to the benefits provided by the highly hydrated, extended, 3D structured polymer chains,^12^ which lead to the high capacity for analyte binding, the ability to suppress nonspecific binding, as well as the possibility of chemical variability. Among branched polymers, the natural biopolymer dextran has become widely used in biosensor applications. Dextran is a branched glucose polysaccharide able to absorb a large amount of water in its native form. A carboxymethylated form of dextran, which enables the introduction of reactive anchor groups, is especially suited for the conjugation of biomolecules as covalent bonds between an aminated surface and the carboxyl groups of the carboxymethyl-dextran (CMD) can be formed using carbodiimide-based linking chemistry.^13,14^ The resulting high concentration of reactive groups provides high capacity of immobilization of biomolecules.^15^ Moreover, the flexibility of dextran chains facilitates the accessibility of binding sites on the immobilized protein. For example, several commercially available SPR sensor chips, which are composed of a glass chip coated with a thin layer of gold functionalized by an additional chemical coating, use CMD self-assembled monolayers (SAMs) as the immobilization matrix.^16−18^ Indeed, the high sensitivity and selectivity of SPR binding assays are enabled by the strong chemical robustness and stability of the CMD matrix,^19^ the high packing density of immobilized proteins, combined with low nonspecific adsorption conferred by the large exclusion volume, the steric repulsion effect, and the reduction of surface interfacial energy of the matrix.^13,20^ However, commercial SPR sensor chips suffer from high cost and limited assay parallelism.^5^

Here we present a new strategy for the fabrication of carboxymethyl-dextran-based protein patterned surfaces. The arrays consist of silicon or glass substrates with patterned bioactive areas composed of a thin film of CMD separated by hydrophobic nonprotein-binding areas. The patterning creates a confinement of the sampling solution within the bioactive areas, which is aimed to confer an array-like feature to the surface. This allows to obtain a detection tool for simultaneous screening of multiple samples and experimental conditions needed for the optimization of bioassays. The fabrication process is compatible with large scale production of biomolecule immobilization surfaces for standard protocols optimization. Our proposed devices were validated using a chemiluminescence assay that exploits an innovative DNA nanoassembly-based approach, that we recently developed,^21^ which was performed on target proteins immobilized on the bioactive areas. Through this assay, we were able to characterize the chemical reactivity of two target proteins toward the dextran matrix of patterned surfaces and to compare it with model SPR surfaces. We found not only a high reproducibility and selectivity of the molecular recognition, but we also found that differences in luminescence signal among the two target proteins were consistent with the results obtained with SPR sensor surfaces. The validation of our devices performed in parallel with SPR assays shows that our arrays are a useful strategy for the implementation of interface protein analysis techniques.

## Results and discussion

**Figure 1:**
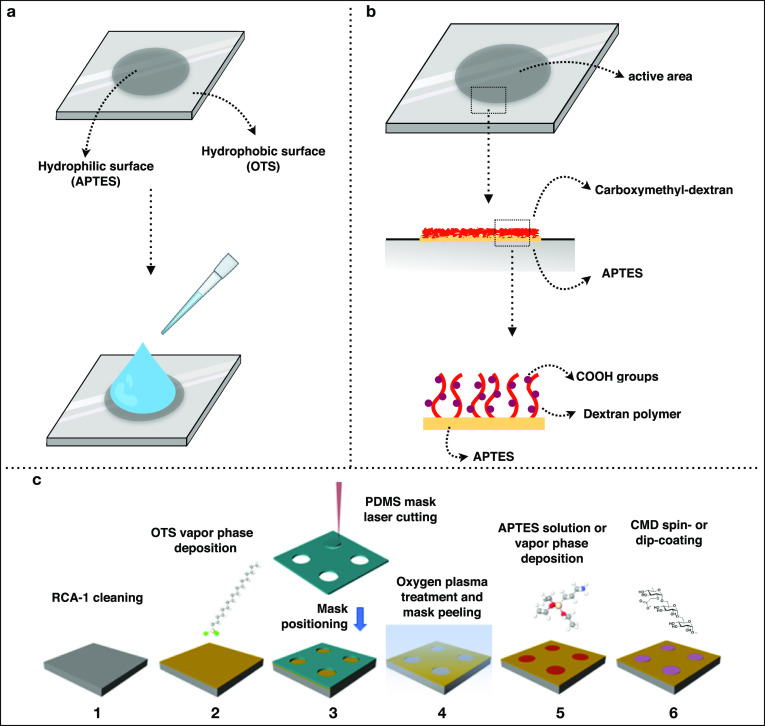
Schematic representation of carboxmethyl-dextran patterned silicon surfaces. (a) Square silicon surfaces (10 × 10 cm) or glass surface (2 × 2 cm) were treated with OTS to create a hydrophobic SAM. Round hydrophilic active areas (1 cm diameter) were created by removal of OTS SAM and subsequent deposition of APTES. The dual feature of the surface allows to confine the sample/reaction drop in the limited active area. (b) Schematic of the composition of active areas. Deposition of APTES created a layer to promote the adhesion of carboxmethyl-dextran brushes. The chains of dextran polymer are functionalized with carboxylic groups. (c) Schematic representation of the fabrication process of carboxymethyl-dextran functionalization. (1) Surface cleaning by RCA-1 solution, (2) OTS vapor phase deposition, (3) PDMS mask fabrication and positioning, (4) oxygen plasma treatment and mask peeling off, (5) APTES solution/vapor phase deposition, (6) CMD spin coating or dip coating.

**Figure 2:**
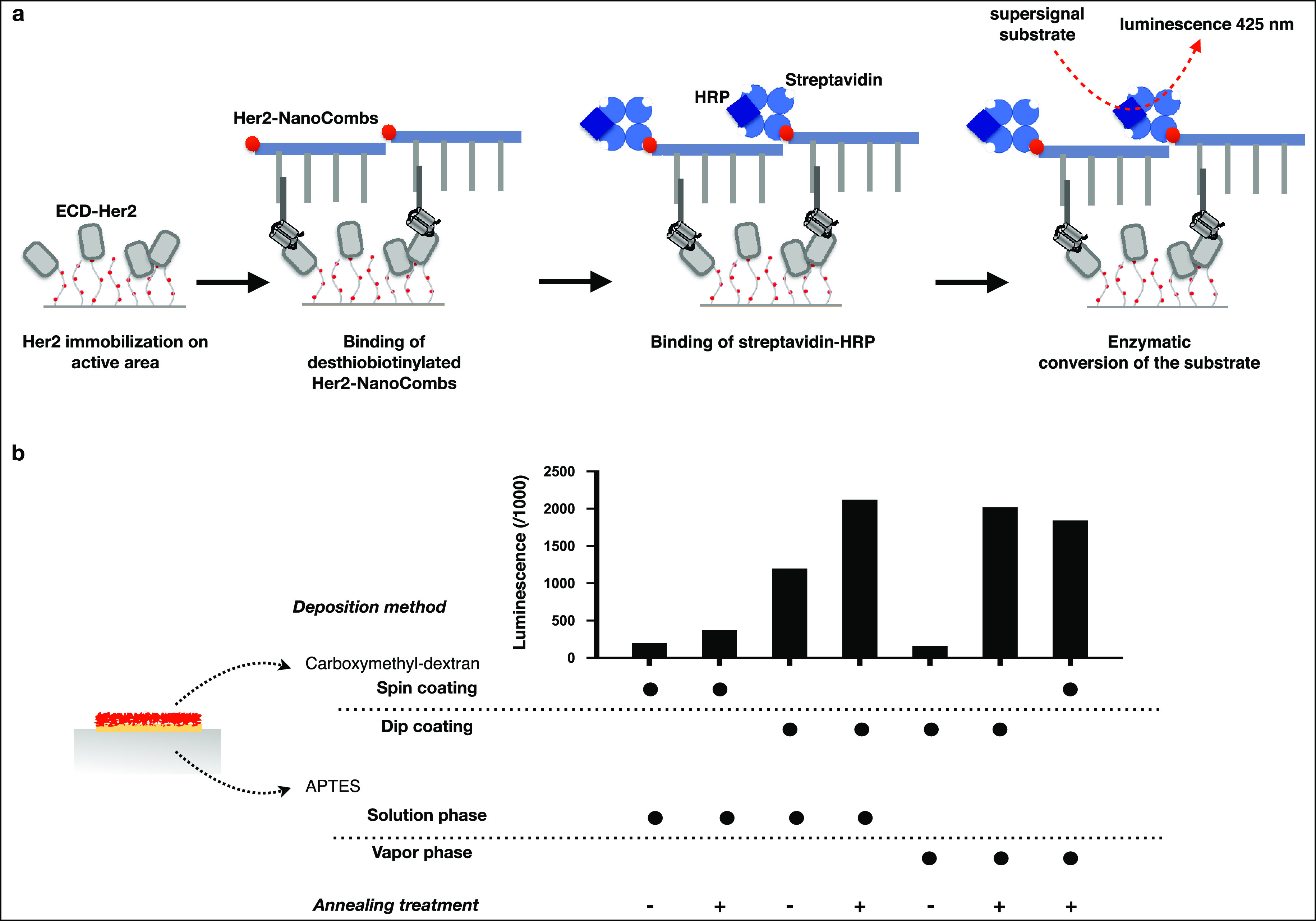
Fabrication parameters of patterned surfaces affect the yield of immobilization of protein of interest in the active area. (a) Schematic of the chemiluminescence assay used to evaluate the yield of protein immobilization on the SiO_2_ surfaces. ECD-Her2 was covalently immobilized on the active area by means of coupling of the amine groups of protein to the carboxylic groups of dextran. Active areas were then treated with DNA nanoassemblies (NanoCombs) preloaded with anti-Her2 affibody (Her2-NanoCombs) and modified with a desthiobiotin. After washing, patterned surfaces were then incubated with streptavidin conjugated with horseradish peroxidase. The enzyme catalyzes the substrate conversion, leading to the luminescence emission at 425 nm. (b) Luminescence values are presented on histogram. Samples differ for fabrication parameters of three steps: deposition of APTES (from vapor phase versus solution phase), annealing treatment after APTES deposition (with versus without), and deposition of carboxmethyl-dextran (by spin coating versus dip coating).

We designed arrays consisting of silicon or glass substrates with patterned bioactive areas that were able to efficiently confine the sampling solution by simply exploiting hydrophilic/hydrophobic patterning of the surface. This bifunctional patterning was obtained by the deposition of (3-aminopropyl)triethoxysilane (APTES) on 1 cm diameter selected areas, surrounded by a SAM of octadecyl trichlorosilane (OTS) (Figure 1a). APTES allows for efficient immobilization of CMD, as demonstrated in several studies,^22,23^ while OTS provides the necessary hydrophobicity for the confinement of water-based sample solutions. APTES areas were then functionalized with a thin film of CMD deposited to obtain the bioactive areas (Figure 1b). The fabrication process involves the use of instruments and techniques compatible with large scale production (Figure 1c). Briefly, the substrates (SiO_2_ or glass coverslip) were cleaned to efficiently remove the organic contaminants and promote the formation of hydroxyl groups required for functionalization with OTS. After the cleaning, the samples were rinsed with deionized (DI) water and stored in a N_2_ inert atmosphere and the deposition of OTS was then accomplished by vapor phase deposition. Suitable process conditions were investigated, including an annealing treatment to promote surface uniformity and hydrophobicity. Then, we proceeded with the deposition of APTES on defined patterned areas. To achieve selective deposition, we used a silicon mask, produced from a silicone foil in which we created holes of the desired dimensions by means of a CO_2_ laser cutter. This method enables fast fabrication of masks with large dimensions, allowing the preparation of tens of samples simultaneously. Moreover, silicone-based masks are reusable and, exploiting the self-adhesion properties of flat silicone, are able to stick to the OTS modified surface. The surface covered with the mask was subjected to an oxygen plasma treatment to promote the removal of OTS on the exposed areas. Afterward, the sample was transferred to a chemical vapor deposition (CVD) chamber or kept in solution incubation for the deposition of APTES, which selectively bound to the areas where hydrophobic OTS SAM was removed. We verified the quality and the reproducibility of OTS/APTES SAM deposition with water contact angle measurements (Figure S1) and atomic force microscopy (AFM) imaging (Figure S2) on SiO_2_ substrates. The water contact angles on APTES areas were different compared to the contact angles on OTS, showing an efficient patterned deposition of APTES SAM. Moreover, water contact angles were measured before and after the patterning process, demonstrating that the mask-based removal of OTS with oxygen plasma treatment effectively removed OTS from the exposed areas and also protected the unexposed areas from the effects of oxygen plasma treatment, hence preserving the hydrophobicity on OTS areas and the selectivity on APTES areas for the subsequent deposition of CMD (Figure S1). Assembly of the SAMs absorbed on the surfaces was also imaged with AFM (Figure S2). The surfaces appeared to be fully covered and, by evaluation of the roughness values, we detected an increase of the surface roughness for both SAMs of OTS and APTES, with respect to the value obtained with cleaned silicon substrate, from 0.09 to 0.19 nm and to 0.21 nm, respectively. The proposed process allows the definition of complex patterning of surface functionalization down to the laser cutting resolution, avoiding the use of more complex and less reliable techniques.^24^ Indeed, many methods for SAM patterning (i.e., microcontact printing, AFM grafting, dip-pen nanolithography) are described in the literature; our alternative approach allowed us to reach the resolution required for the device, ensuring at the same time a much faster and easily scalable method. Moreover, it allows for selective functionalization of surfaces with two different SAMs in a few simple steps, without affecting their quality and cleanliness, as demonstrated by the AFM and contact angle analysis performed. Finally, we deposited the CMD on APTES functionalized areas. We tested and characterized two different deposition methods, such as dip coating and spin coating. Although dip coating is the preferred approach for complex patterns, resulting in very precise separation between hydrophobic and hydrophilic area, spin coating results in faster and more reliable CMD deposition, including thickness control and uniformity on the deposited film.^25,26^ The CMD matrix was visualized by AFM imaging as molecular aggregates revealed by the increase of surface roughness (Figure S3a,b). Although overall roughness of surface, determined in areas of 5 × 5 μm, obtained by spin coating deposition was higher than that obtained by dip coating deposition (Figure S3a), we detected a comparable local roughness, determined in some of smaller areas (1 × 1 μm) (Figure S3b). Moreover, we observed a higher reproducibility of the spin coating deposition compared to dip coating deposition. Both AFM imaging and optical profilometer analysis of CMD showed that the height of the dextran deposition obtained by spin coating is in the range of 20–40 nm, in agreement with previously reported data.^27^ Instead, dip coating deposition led to a thicker CMD layer (Figure S3c). The higher molecular weight (MW) of CMD entails longer branching chains and higher layer thickness, which leads to a higher protein immobilization potential. On the contrary, a high-density dextran matrix can face several challenges such as matrix swelling, because of chain repulsion and matrix expansion,^28,29^ and steric hindrance. Consequently, the penetration capability for analyte to reach all immobilized protein available decreases and the protein/analyte equilibrium state is achieved at longer times,^28^ leading to the reduction of detection sensitivity. For this reason, a trade-off between these two events, by the selection of an intermediate MW (20 kDa) and thickness of the dextran layer (20–40 nm), was preferred in the surface fabrication to ensure optimal results in biomolecule binding assays. To understand and characterize how biomolecules interact with our functionalized SiO_2_ surfaces and to evaluate the protein immobilization yield resulting from each of the different fabrication processes (APTES, solution vs vapor phase deposition, plus vs minus annealing treatment; CMD, dip vs spin coating), we performed a bioassay that we recently implemented in the development of a nonmicroscopy-based method for ensemble analysis of membrane protein nanodomains, named NanoDeep.^21^ Briefly, the DNA nanoassembly, termed NanoComb, consists of a double-stranded backbone with four single-stranded DNA sequences (prongs) that protrude from the backbone at regular intervals. The first prong is preloaded with an oligonucleotide-conjugated binder specific for a target protein. In our workflow, the target protein was the extracellular domain of the human epidermal growth factor receptor 2 (ECD-Her2), which was immobilized through amine coupling on active areas of the CMD-based patterned surfaces. The amine coupling chemistry (EDC/NHS) used to activate the CMD layer for biomolecule immobilization, enabled at the same time covalent grafting of CMD to the surface, ensuring stronger stability of the interaction between APTES and the CMD, but also preserving carboxylic groups of CMD for being exploited to the immobilization of the target biomolecule. Then the surfaces were treated with NanoCombs preloaded with anti-Her2 affibody (hereinafter Her2-NanoCombs), previously shown to have a high affinity for its target.^21^ NanoCombs were modified with desthiobiotin at the 3′ end of the backbone. After incubating with a streptavidin conjugated with horseradish peroxidase, a specific substrate was catalyzed by the peroxidase and converted into a luminescence signal (Figure 2a). The analysis of luminescence data revealed that the surface sample preparation affects the yield of immobilization of the selected protein. As shown in Figure 2b, the lowest luminescence signal, if considering both the processes with and without an annealing treatment, was observed for surfaces obtained by solution phase deposition of APTES, followed by spin coating deposition of CMD. A possible explanation of the low yield of protein immobilization resulting from the combination of these two processes can be found in the observation that APTES, deposited in the solution phase, results in a SAM with low homogeneity. This can have a negative effect on the subsequent deposition of CMD, which impacts more in a thin layer deposition, as applied in the spin coating method, rather than a thick layer deposition, as applied in the dip coating method. Moreover, we detected a positive effect of the annealing treatment, performed after the APTES deposition, in all the conditions that we tested. The vacuum thermal process, entailed by the annealing treatment, is capable to promote the selective removal of APTES and OTS molecules that are physiosorbed on the surface but not chemically bound, enhancing the subsequent reproducible deposition of the CMD.^30,31^ Moreover, it has already been demonstrated that the thermal treatment at the temperature of 120 °C is crucial to promote the covalent binding of APTES molecules to the hydrophilic surface.^32^ We found that, among the different samples analyzed, the surfaces obtained by solution phase deposition of APTES, followed by annealing, and dip coating deposition of CMD, or vapor phase deposition of APTES, followed by annealing treatment, and spin or dip coating deposition of CMD yielded to highest luminescence values (Figure 2b), indicating that the highest level of protein immobilization was reached with these fabrication workflows. Although dip coating is the preferential approach for complex patterns, spin coating resulted in faster and more reliable CMD deposition, including thickness control on the deposited films. For this reason, even if a comparable luminescence signal was obtained for the three above-mentioned SiO_2_ surfaces, we selected the one achieved by spin coating for further processing and characterization.

**Figure 3:**
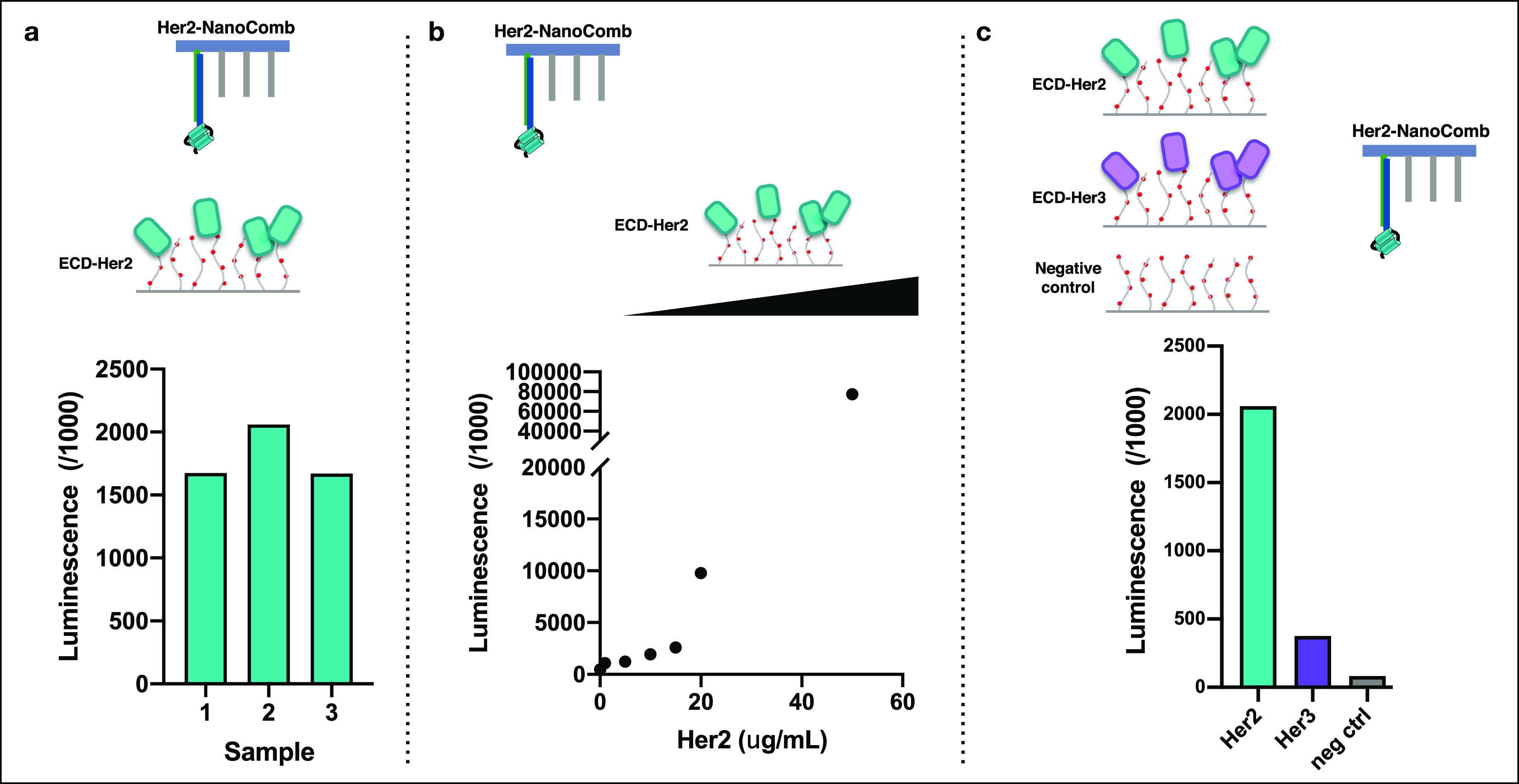
Characterization of the selected SiO_2_ surface (APTES, vapor phase/annealing, +/DEXTRAN, spin coating). (a) Evaluation of the reproducibility of protein immobilization. Three SiO_2_ sample surfaces were processed as replicates of the same experiment, which entails first the immobilization of ECD-Her2 on patterned surfaces by means of amine coupling and then testing with a chemiluminescence assay to determine the level of protein immobilization, as previously described. (b) Evaluation of proportionality between amount of protein used for immobilization and yield of immobilization. Different amounts of ECD-Her2 (from 1 to 50 μg/mL) were immobilized on different patterned surfaces. The chemiluminescent assay revealed a correlation between protein concentration used in the immobilization step and readout signal. (c) Evaluation of specificity of luminescence readout. Three surfaces presenting ECD-Her2, ECD-Her3, and without any protein as a negative control were created. Chemiluminescence signal showed that Her2-NanoComb bound specifically to the surface presenting its target (ECD-Her2) and that there was minimal background binding to the surface that did not present the correct target protein (ECD-Her3) or to the negative control.

We treated three independent samples of the selected SiO_2_ surfaces with the same amount of the Her2 protein. A similar chemiluminescence signal obtained by the DNA nanoassembly-based assay indicated the same yield of protein immobilization, confirming the reproducibility and stability of the surface functionalization (Figure 3a). We observed a correlation between amount of protein used for immobilization and the readout signal, which reflects the yield of immobilization (Figure 3b). Moreover, we found that the readout signal is specific to the immobilized protein (Figure 3c), indicating that the fabrication processes used to deposit CMD preserved its features of high chemical specificity and, importantly, minimal nonspecific binding.

**Figure 4:**
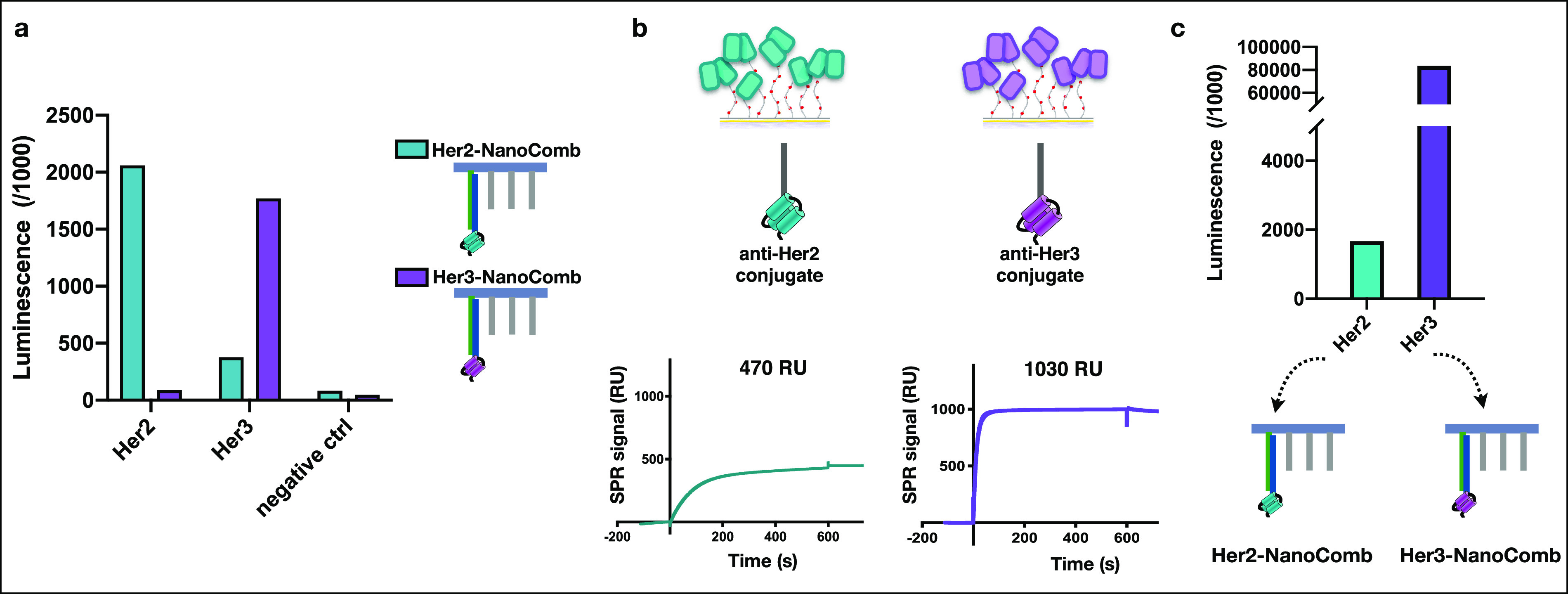
Validation of specificity of the luminescence readout on CMD patterned SiO_2_ surfaces. (a) Cross-reactivity test: patterned SiO_2_ surfaces presenting Her2, Her3 (immobilized at a concentration to obtain the same NanoComb binding level, as tested in Figure S4), and without any protein, as a negative control, were treated with Her2- and Her3-NanoCombs (for Her2-NanoComb same as in Figure 3c). The chemiluminescence assay was performed, and signals obtained from the two different NanoCombs on surfaces functionalized with the same target revealed the absence of cross-reactivity. (b and c) Comparable difference in chemical reactivity of two target proteins toward the dextran matrix of patterned surfaces and of model SPR surfaces leads to comparable different yield of immobilization. (b) Two different target proteins (Her2 and Her3) were covalently attached to two distinct SPR sensor surfaces, functionalized with a CMD matrix. The amount of immobilized protein was estimated from the SPR signal of binder-oligo conjugate specific for each protein and injected at 10-fold the *K*_D_ for their respective target. (c) Same target proteins (Her2 and Her3) were covalently immobilized on two distinct patterned SiO_2_ surfaces at 10 μg/mL. NanoCombs specific for each surface were incubated, and the amount of immobilized protein was verified with the chemiluminescence readout. The difference of luminescence signal among the two target proteins is comparable to that obtained with SPR sensor surfaces.

To further prove the specificity of detection performed on our device, as a result of absence of nonspecific binding of proteins during the immobilization and of binder-oligo conjugates during the binding assay, we performed a cross-reactivity test, in which binding of Her2- and Her3-NanoCombs was tested over SiO_2_ surfaces presenting ECD-Her2 and ECD-Her3. The luminescence signal was observed only when NanoCombs were incubated over the surfaces presenting their respective target proteins (Figure 4a). Next, we correlated the SPR signal obtained on SPR sensor chip with the luminescence readout obtained on our CMD-based arrays (Figure 4b,c). We immobilized two different target proteins (ECD-Her2 and ECD-Her3) on CMD-based SPR sensor chips (CM5 chip) using the same concentrations for the two proteins in the amine coupling reaction. Then, we recorded the binding of the binder-oligo conjugates specific for each of the two proteins, by injecting them over the SPR sensor surface at saturating concentrations. We observed different binding levels of the binder oligo conjugates for their respective target proteins (Figure 4b), attributed both to different immobilization yields of the target proteins by amine coupling on the CMD matrix of the SPR sensor chip, and to the specific interactions between binders and the target proteins. In parallel we performed the same binding test on our CMD-based patterned surfaces. We immobilized the two target proteins on two distinct active areas of the SiO_2_ surface and then we ran the chemiluminescence assay by using NanoCombs functionalized with the binder-oligo conjugates used in the SPR experiment (Her2- and Her3-NanoComb) (Figure 4c). We found a good correlation between the luminescence signal and the SPR binding level for each binder-oligo/target protein pair. Together, these results showed that our CMD-based arrays have a protein immobilization capability and detection performance of their respective binders that are comparable to commercial CMD-based SPR sensor chips.

**Figure 5:**
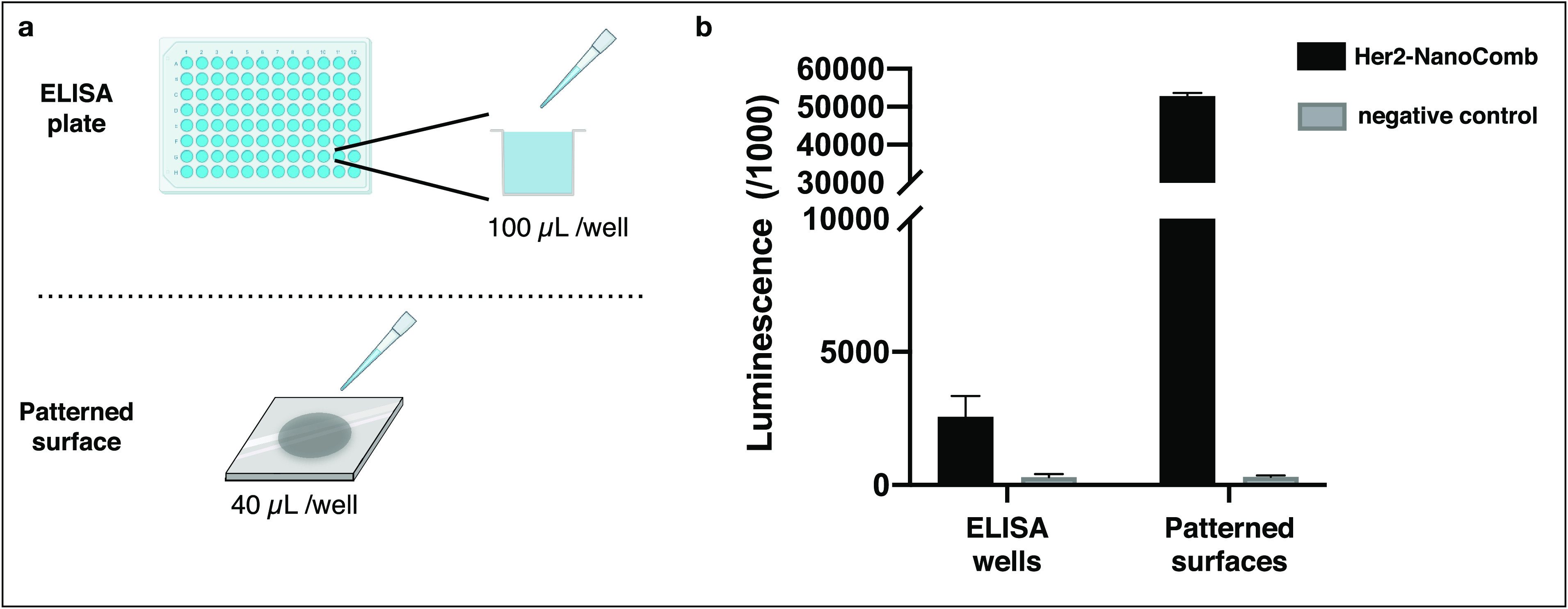
Binding assay on patterned surfaces exhibits higher performance compared to standard plate-based assay. (a) His-tagged ECD-Her2 was immobilized in parallel on patterned surfaces and on Nickel coated 96-well plate, commonly used for ELISA and other standard plate-based binding assays. (b) Chemiluminescence assay performed in triplicate on both formats showed that patterned SiO_2_ surfaces allow the immobilization of higher amounts of target protein in lower volume if compared to a 96-well plate. Volumes used for target protein immobilization and subsequent steps of the chemiluminescence assay are reduced in a patterned surfaces assay if compared to the 96-well plate assay.

Moreover, we compared biomolecular interaction analyses performed on our CMD-based patterned surfaces with standard, commonly used, ELISA plate-based assays (Figure 5). Our CMD-based chips allow for the immobilization of higher amounts of target protein in lower volume compared to 96-well plates. Higher detection signals in reduced volumes of the chemiluminescence assay also enable the possibility of future miniaturization of the system.

**Figure 6:**
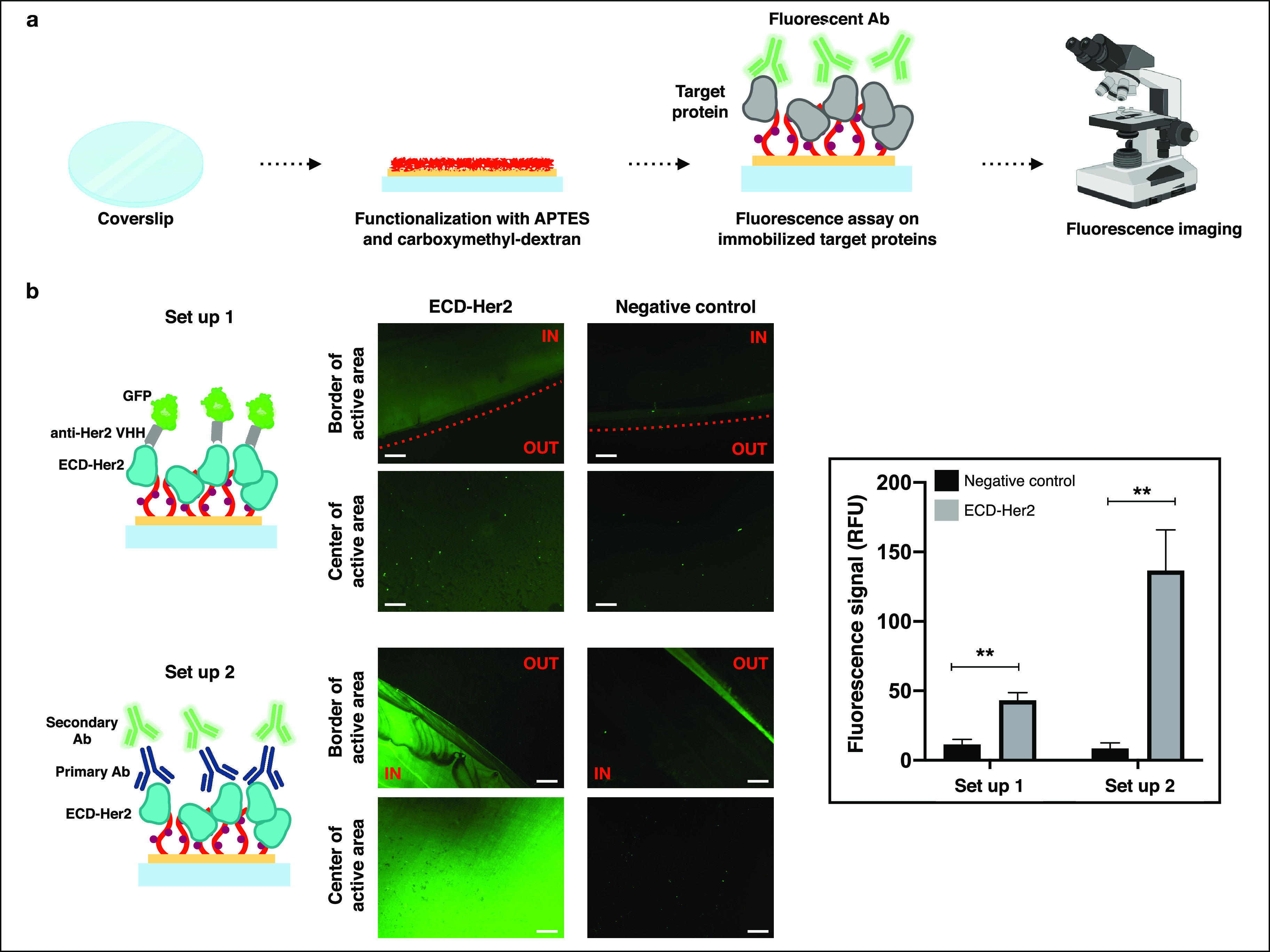
Carboxymethyl-dextran functionalization of glass coverslip allows use of fluorescence imaging as a readout of binding assays. (a) Schematic representation of fluorescent assay performed on carboxymethyl-dextran functionalized surfaces. ECD-Her2 was immobilized on cover glass, functionalized with CMD, by an amine coupling reaction. Then two different fluorescence assays set up were performed, and the fluorescence signal was observed on fluorescence microscopy. (b) In set up 1 a nanobody (VHH) specific for ECD-Her2 and labeled with GFP was incubated over the surface. In set up 2 a first incubation with a primary Ab specific for ECD-Her2 was followed by the incubation with secondary Ab labeled with Alexa488 fluorophore. In set ups 1 and 2 the fluorescence signal was acquired in triplicate with a fluorescence microscope. Quantification of the signal is shown in the histogram. **P ≤ 0.05. Scale bars: 100 μm.

We applied the selected fabrication process developed on the SiO_2_ surface for the functionalization of a cover glass substrate. By replicating on a transparent substrate the process optimized on SiO_2_ samples, we aimed to produce surface samples that can be used in microscopy and imaging applications (Figure 6). Immobilization of ECD-Her2 as a target protein on glass surfaces was evaluated by standard immunoassays (Figure 6a). For both the immunoassays performed, we observed a specific binding of ECD-Her2 through amine coupling chemistry of the immobilization step and we did not detect any presence of nonspecific binding of nanobody and antibodies used for the fluorescence detection. Moreover, we proved that the biomolecules immobilization/interaction only occurred on the bioactive areas of the surface (Figure 6b).

**Figure 7:**
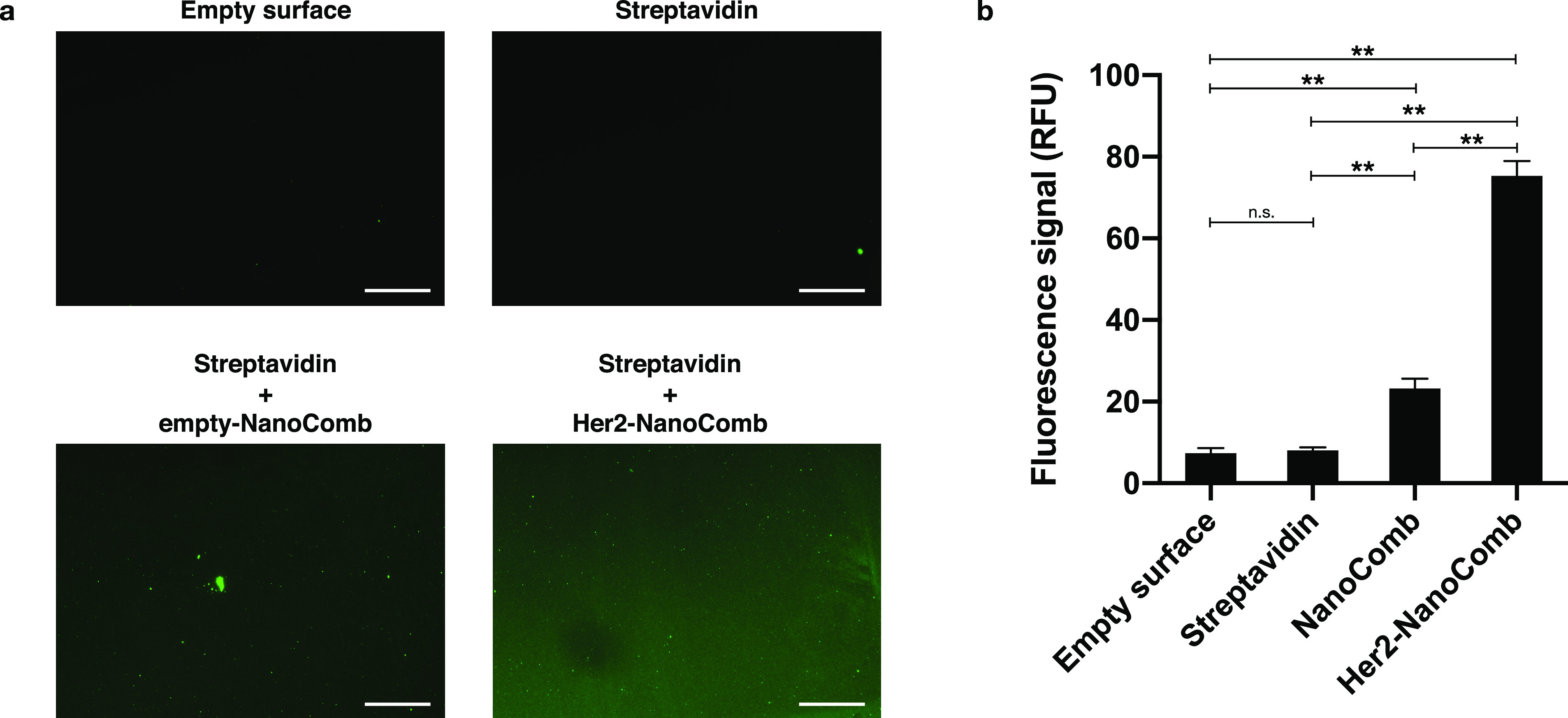
Carboxymethyl-dextran functionalization of glass coverslip allows the immobilization of Her2-NanoCombs for specific detection of ECD-Her2. Desthiobiotin-Her2-NanoCombs were immobilized on glass CMD-based patterned surfaces through streptavidin molecules previously attached by means of amine coupling. Empty surface, surfaces with immobilized streptavidin, and a surface with streptavidin with anchored empty-NanoComb were used as negative controls. Binding of ECD-Her2 was determined through a primary Ab specific for ECD-Her2, followed by the incubation with a secondary Ab labeled with an Alexa488 fluorophore. (a) Fluorescence images of the four samples. Scale bars: 100 μm. (b) Quantification of the fluorescence signal, obtained in triplicate, is shown in the histogram. **P ≤ 0.05.

Finally, we tested the capability of our CMD-based patterned glass surfaces to conversely immobilize the Her2-NanoCombs and then perform the recognition of ECD-Her2. Also, in this case, we observed a specific binding between ECD-Her2 and Her2-NanoCombs, detected through an anti-Her2 primary Ab and a secondary fluorescence Ab (Figure 7), compared to all the negative controls (empty surface, immobilized Streptavidin, biotinylated NanoComb not functionalized with anti-Her2 affibody).

## Conclusion

In this work, we developed a fabrication procedure for patterned functionalization of bioanalytical platforms. Although functionalization of surfaces with dextran has been already described in the literature as a strategy to obtain an immobilization matrix, here we showed the development of CMD patterned surfaces. We combined standard, easy, and cost-effective fabrication techniques, such as vapor phase deposition, laser cutter, oxygen plasma treatment, and dip or spin coating, to create SiO_2_ and glass substrates with delimited CMD-based bioactive areas for efficient and specific immobilization of proteins. We showed that biomolecular interactions observed with our surfaces correlate with results obtained in CMD-based SPR sensor chips, allowing the translation of the highest quality data on characterization of biomolecular interactions, such as with the gold standard SPR technique, to simple assays feasible on our CMD-based chips. The methodology is straightforward, cheap, and provides the means to create a valid alternative for biomolecular interaction assays. Moreover, the validation of our surfaces in parallel with SPR assays is advantageous compared to other surface and interface protein analysis techniques, since we can directly translate the information-rich content of an interaction, together with high sensitive and high accurate detection results of real-time binding events, on our CMD-based chip, taking advantage of a cost-effective, straightforward, and higher throughput assay device.

## Methods

### Fabrication of CMD Patterned Surfaces

#### Cleaning of Silicon Substrates

Silicon wafers were cut into pieces (approximately 3.0 × 4.5 cm) and processed with an appropriate cleaning and surface activation. Cleaning of the silicon substrates was performed by keeping them in a piranha solution (3:1; H_2_SO_4_/H_2_O_2_) for 15 min, to efficiently remove the organic contaminants and promote the formation of the hydroxy group for the following functionalization step with OTS. The substrates were then carefully rinsed with DI water and dried under a stream of nitrogen (N_2_) gas. After the cleaning step, oxygen plasma treatment by reactive-ion etching (RIE) for 2 min (40 W, Bias 100 V) was employed to activate the OH group on the surface.

#### Cleaning of Glass Substrates

Glass substrates (22 × 22 mm) were first cleaned in soap water, rinsed in DI water, and finally immersed in a freshly prepared RCA-1 solution (5:1:1; H_2_O/H_2_O_2_/NH_3_) for 20 min at 70 °C. Then they were washed with DI water, acetone, isopropyl alcohol (IPA), and dried by a N_2_ stream. Subsequently, glass substrates were further cleaned with oxygen plasma treatment by RIE for 2 min (40 W, Bias 100 V) to allow the activation of the silanol groups on the surface.

#### Laser-Patterned PDMS Mask

A silicone mask with a desired pattern was prepared by means of a laser cutting methodology, directly on a silicone foil (ELASTOSIL Film 2030, 200 μm thickness, Wacker Chemie AG). We used a commercial CO_2_ laser plotter (Versa Laser System, Model VLS3.50, Universal Laser System, Ltd.) set with the following parameters: maximum power 25 W, maximum pulses per inch (PPI) 1000, and scanning speed ranging from 0.25 to 25 mm/s.

#### OTS/APTES Functionalization Process

A three step process was performed to create the patterned functionalization of the surface: (1) growth of OTS SAM by vapor deposition; (2) selective removal of OTS through the silicon foil mask, by oxygen plasma by RIE; (3) APTES deposition on plasma exposed regions. (1) OTS was deposited on samples through vapor deposition process (in a static vacuum) for 4 h at room temperature (RT). The annealing process, consisting of incubation of the samples for 2 h at 120 °C in a continuum vacuum, promoted the removal of unreacted molecules adsorbed on the surface, resulting in a more homogeneous and hydrophobic SAM. (2) The laser-patterned silicon mask was placed on samples, which were then exposed to oxygen plasma treatment (40 W, Bias 120 V) to remove OTS on the exposed areas. (3) APTES (99%, Sigma-Aldrich) was deposited on samples through incubation in a solution of ethanol (0.1% v/v) for 2 h or by a vapor deposition process (in a continuum vacuum) in a glass chamber. Then, 300 μL of silane was injected in a glass Petri dish on the underside of the chamber. The chamber was kept in a vacuum and heated at 50 °C for 4 h. After this time, the samples were placed in a vacuum oven for 2 h at 120 °C.

#### CMD Deposition

We prepared the solution by dissolving the CMD (Sigma-Aldrich, MW = 20 kDa) in DI water at variable concentrations from 1 to 10% in weight; the solution was then stirred overnight to allow the complete and homogeneous dissolution. For the spin coating process, a 5% solution was used; the final thickness could be modulated by changing the spin speed. Ellipsometry data showed high rotational speed dependence on the CMD layer thickness comparing the thickness values at 1000 and 2000 rpm. However, varying the rotational speeds to 3000 and 4000 rpm did not cause significant differences in the CMD layer thickness.^13^ We chose 2000 rpm as optimal rotational speed in our deposition protocol.

### Characterization of CMD Patterned Surfaces

#### AFM

Surface morphologies and roughness were characterized through AFM imaging, using a MFP-3D atomic force microscope (Asylum Research) in tapping mode in air using a silicon cantilever (Mikromasch, NSC19/AL BS) with a nominal tip radius of ∼8 nm and a spring constant of *k* = 0.6 N/m (*f* ≈ 65 kHz). The 512 × 512 pixels images on a 5 × 5 μm area on the chip surface were acquired; two scans were acquired for each surface, each one from a separate chip. The Gwyddion software was used for the quantitative analysis of AFM images.

#### Contact Angle (CA)

Contact angle measurements were carried out on a DataPhysics OCA 15Pro optical instrument (DataPhysics Instruments GmbH, Germany) at ambient temperature by placing 2 μL of Milli-Q water onto the sensor surface. The average CA values were obtained by measuring five different positions on each sample surface.

#### Optical Profile Images

3D optical profiles were obtained using a Profilm3D optical profilometer (Filmetrics Inc., USA).

### Chemiluminescence Assay

ECD-Her2 and ECD-Her3 (ACROBiosystems) (Sino Biological) proteins were immobilized on active areas of our microfabricated SiO_2_ CMD-based chip through covalent amine coupling. Briefly, 10 min of incubation of a mixture of 1-ethyl-3-(3 dimethylaminopropyl)carbodiimide hydrochloride (EDC) and *N*-hydroxysuccinimide (NHS) (Cytiva) was used to activate the carboxylic groups of CMD. Proteins were diluted in a Na acetate pH 4.0–4.5 buffer (Cytiva) and incubated for 20 min over the activated surfaces. Finally, not-reacted carboxylic groups were blocked with a solution of ethanolamine hydrochloride-NaOH pH 8.5 (Cytiva) for 10 min. Patterned CMD-based surfaces presenting ECD-Her2 or ECD-Her3 were then treated with Her2- and Her3-NanoCombs modified with desthiobiotin at the 3′ end of the backbone for 2 h at RT, followed by washing with phosphate buffered saline (PBS) + 0.05% Tween20. Surfaces were then incubated with streptavidin conjugated with horseradish peroxidase (Thermo Fisher Scientific) for 20 min at RT, followed by washing. The SuperSignal enzyme-linked immunosorbent assay Pico chemiluminescent substrate (Thermo Fisher Scientific) was added, and substrate conversion catalyzed by horseradish peroxidase was performed for 1 min at RT. Luminescence at 425 nm was measured within 5 min after the end of the reaction with a Varioskan Lux plate reader (Thermo Fisher Scientific).

### ELISA Plate-Based Chemiluminescent Assay

Pierce Nickel Coated Plates (Thermo Fisher Scientific) were used to immobilize His-tagged ECD-Her2 proteins (1 h incubation). After washing with PBS + 0.05% Tween20, we performed a chemiluminescent assay as described above for CMD-based patterned surfaces.

### Fluorescence Assay

CMD-based glass coverslips were processed to perform ECD-Her2 covalent amine coupling, as described above for SiO_2_ CMD-based surfaces. Two fluorescence assay set ups were performed. Set up 1: surfaces were incubated with a camelid Nanobody (VHH) specific for ECD-Her2 and coexpressed with Green Fluorescence Protein (GFP) (gift of Prof. Ario de Marco, University of Nova Gorica), for 2 h at RT, followed by washing with PBS + 0.05% Tween20. Set up 2: surfaces were incubated with a primary antibody (Her2 Recombinant Rabbit Monoclonal Antibody, Thermo Fisher Scientific) for 2 h at RT and, after washing with PBS + 0.05% Tween20, with a secondary fluorescent antibody (Donkey anti-Rabbit IgG Secondary Antibody, Alexa Fluor 488, Thermo Fisher Scientific) for 1 h at RT, followed by washing. The fluorescent signal was acquired using a Zeiss Axio Imager.M2 microscope with 10× magnification and quantified in the digital images with the ImageJ program. Three different images of the same region of interest (ROI) were included in the analysis. For the streptavidin-NanoCombs experiment, streptavidin molecules were covalently immobilized by means of amine coupling as previously described. Desthiobiotin-NanoCombs were then incubated over the surface for 1 h at RT.

### SPR Assays

A Biacore T200 instrument and related reagents (Cytiva) were used to perform all SPR experiments. ECD-Her2/ECD-Her3 proteins were immobilized on different flow cells of a CM5 sensor chips via amine coupling reactions, according to the manufacturer’s instructions. Binding tests of anti-Her2 and anti-Her3 binder-oligo conjugates were performed by a saturating concentration of analytes in running buffer (HBS-EP+).

### Statistical Analysis

Statistical analysis was carried out with GraphPad Prism (version 8.2.1). Statistical significance was determined by performing a two-tailed Student’s *t* test. *P* ≤ 0.05 was considered statistically significant.

## Supporting Information Available

Characterization of the OTS and APTES SAMs by means of water contact angle, characterization of the OTS and APTES SAMs by means of AFM imaging, characterization of CMD layer, and Her2- and Her3-NanoCombs binding test on different concentrations of immobilized Her2 and Her3 respective targets (PDF)

## ABBREVIATIONS

CMD: carboxymethyl-dextran
SPR: surface plasmon resonance
ELISA: enzyme-linked immunosorbent assay
SAM: self-assembled monolayers
APTES: (3-aminopropyl)triethoxysilane
OTS: octadecyl trichlorosilane
DI: deionized
CVD: chemical vapor deposition
AFM: atomic force microscopy
MW: molecular weight
EDC: 1-ethyl-3-(3 dimethylaminopropyl)carbodiimide hydrochloride
NHS: *N*-hydroxysuccinimide
ECD: extracellular domain
Her2: human epidermal growth factor receptor 2
Her3: human epidermal growth factor receptor 3
RIE: reactive-ion etching
PDMS: polydimethylsiloxane, PPI, pulses per inch
RT: room temperature
PBS: phosphate buffered saline
VHH: single variable domain on a heavy chain
