# Supplementary material for: Patterned Carboxymethyl-Dextran Functionalized Surfaces Using Organic Mixed Monolayers for Biosensing Applications
Source: ACS Appl Bio Mater. 2022 Jun 25;5(7):3310–9. doi: 10.1021/acsabm.2c00311 (PMC9297292; doi:10.1021/acsabm.2c00311)
Supplement: Supplementary file 1 — mt2c00311_si_001.pdf [file mt2c00311_si_001.pdf]

Supporting Information

Patterned carboxymethyl-dextran functionalized surfaces using organic mixed monolayers for biosensing applications

Elena Ambrosetti[1], Martina Conti[2], Ana I. Teixeira[1], Simone Dal Zilio[2]*

AUTHOR ADDRESS

[1]Karolinska Institutet, Department of Medical Biochemistry and Biophysics, Stockholm, Sweden

[2]CNR-IOM, Istituto Officina dei Materiali-Consiglio Nazionale delle Ricerche, Basovizza, 34149 Trieste, Italy

*Corresponding author: dalzilio@iom.cnr.it

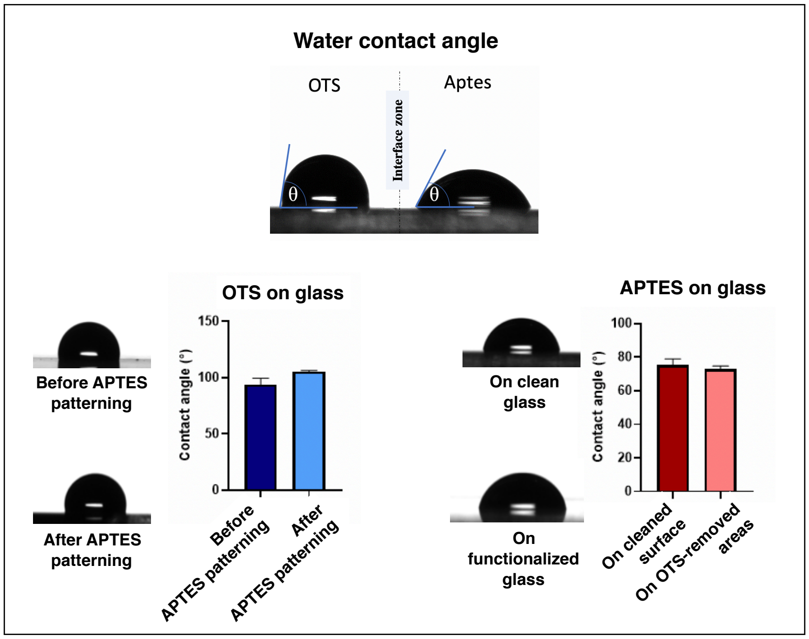

Figure S1. Characterization of the OTS and APTES SAMs by means of water contact angle. Water contact angle at the interface region between OTS and APTES (top). No significant variation on the contact angle values of the OTS SAM deposited on glass before and after the treatment with oxyplasma and APTES deposition demonstrated that the process used for APTES patterning preserve the hydrophobicity feature of OTS SAM (bottom, left). APTES deposited on patterned areas where OTS was removed resulted in similar contact angle value obtained when deposited on cleaned glass (bottom right).

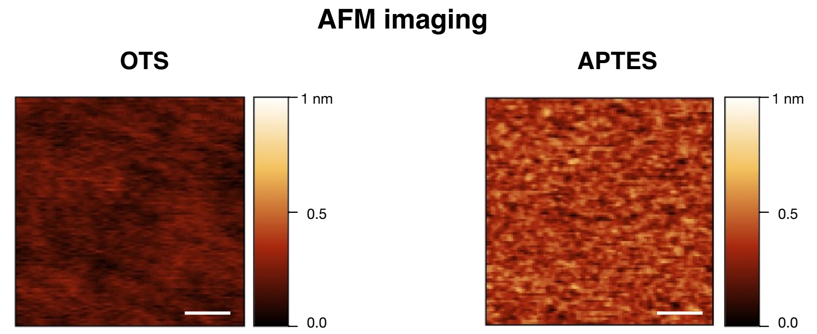

	RMS Roughness (nm)
**Cleaned SiO_2 surface**	0.09
**OTS** after APTES patterning	0.19
**APTES** on OTS-removed areas	0.21

Figure S2. Characterization of the OTS and APTES SAMs by means of AFM imaging. AFM images of OTS and APTES SAM on silicon at the end of the APTES patterning process (OTS deposition, mask-based oxygen plasma removal of OTS and APTES deposition). RMS roughness values of SAMs are shown in the underlying table. Area of images: 1 x 1 μm. Scale bars:

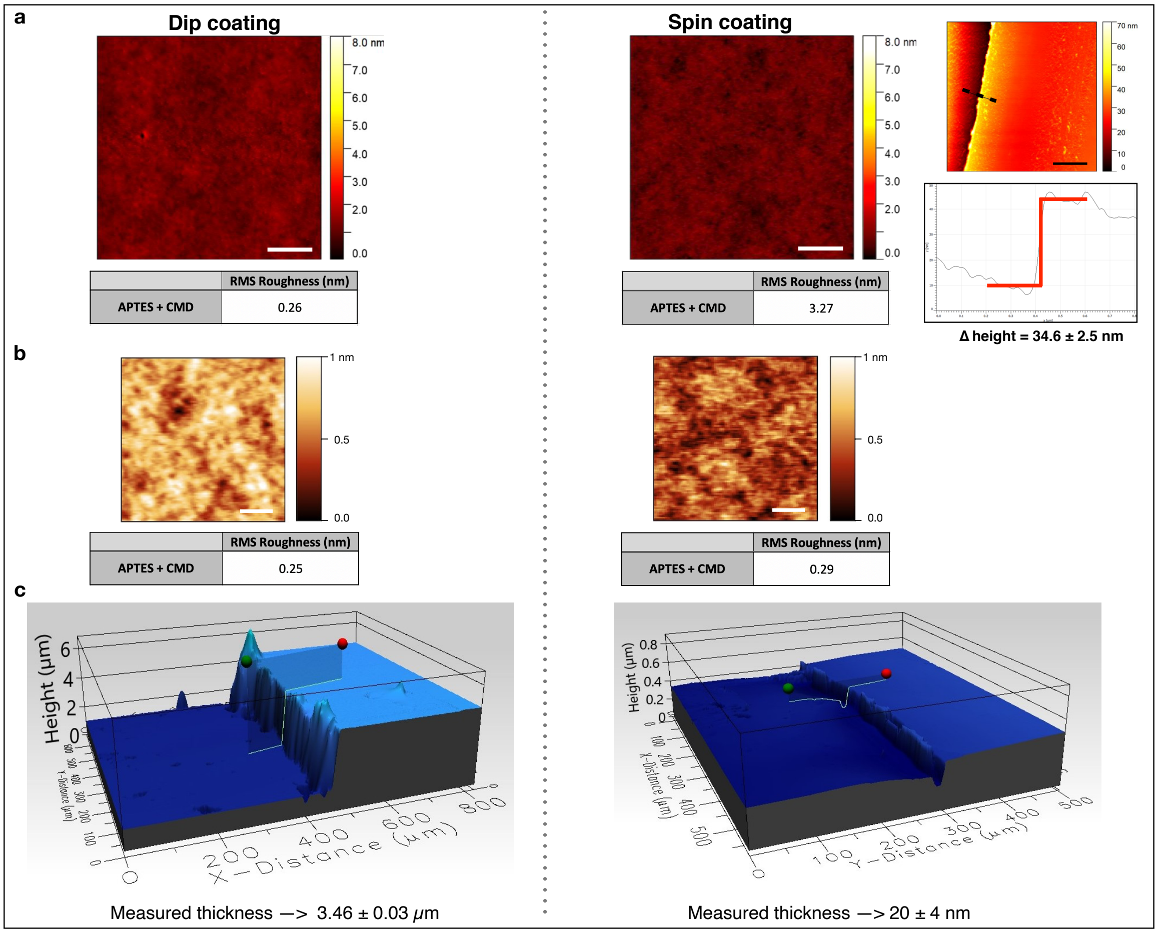

Figure S3. Characterization of CMD layer. (a, b) AFM map and respective roughness values of CMD on SiO_2 deposited by dip coating (left) and spin coating (2000 rpm) (right). (a) Area of images: 5 x 5 µm. Scale bars: 1 µm. n = 10. (b) Area of images: 1 x 1 µm. Scale bars: 200 nm. n = 4. For the sample obtained with spin coating a section analysis (red line on the image) has been performed. (c) Optical profilometer map at the CMD coated region produced by dip coating (left) or spin coating (right).

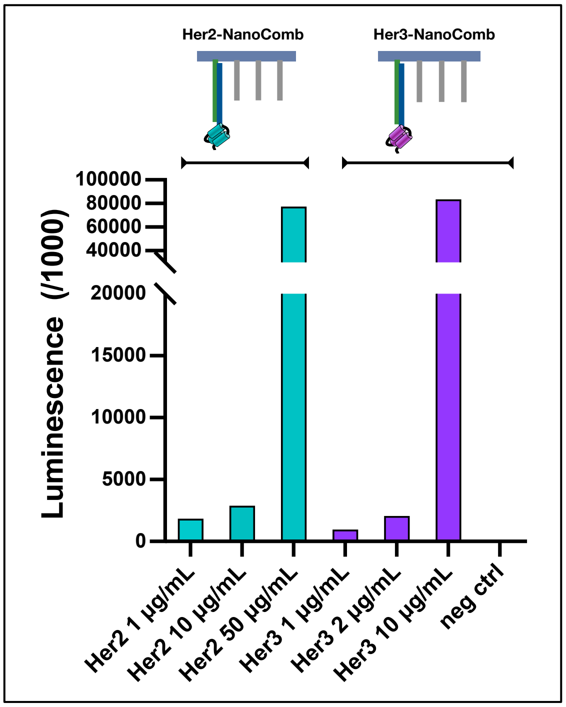

Figure S4. Her2- and Her3-NanoCombs binding test on different concentrations of immobilized Her2 and Her3 respective targets. Different amounts of ECD-Her2 (from 1 to 50 µg/mL) and of ECD-Her3 (from 1 to 10 µg/mL) were immobilized on patterned SiO_2 surfaces. Chemiluminescent assay displayed a different concentration dependency of the signal between the two immobilized proteins.
